# Predicting the development of acute‐on‐chronic liver failure

**DOI:** 10.1002/ueg2.12482

**Published:** 2023-11-03

**Authors:** Maura Morrison, Florent Artru

**Affiliations:** ^1^ Institute of Liver Studies King's College Hospital London UK; ^2^ Department of Inflammation Biology School of Immunology and Microbial Sciences King's College London London UK; ^3^ Liver Disease Unit Rennes University Hospital Rennes France; ^4^ NUMECAN Institute INSERM U1241 and University of Rennes Rennes France

**Keywords:** acute‐on‐chronic liver failure (ACLF), acute decompensation (AD), cirrhosis, liver transplantation

Acute‐on‐chronic liver failure (ACLF), as per the European Association for the Study of the Liver (EASL), is a syndrome that develops in patients with acute decompensation of cirrhosis (AD) which is characterised by severe systemic inflammation, immune dysfunction, organ failures and high short‐term mortality. According to the number of organ failures and dysfunction, defined with specific thresholds for each organ system (liver, coagulation, brain, renal, respiration and circulation), patients are categorised into distinct ACLF grades of increasing severity. Hence, ACLF grade 1 corresponds to a patient with a single kidney failure or a non‐kidney failure with kidney or brain dysfunction), grade 2 is characterised by two organ failures and grade 3 is marked by three organ failures or more. In patients with severe course of ACLF, belonging to grades 2 and 3, the 3‐month mortality escalates to a range of 70% to more than 90%.^1^ Over the past decade, significant efforts have been made to understand the natural history and pathogenesis of the syndrome, standardise clinical management and access to liver transplantation for these patients.^1^ While critical, it is more recently that researchers have tried to identify individuals with AD showing a higher risk of developing ACLF. The PREDICT study identified that AD could follow three different clinical courses over the subsequent 3 months; a ‘pre‐ACLF’ group that develop ACLF, an unstable decompensated cirrhosis group requiring further hospital admission and a stable compensated cirrhosis group with no further admission.^2^ It is the ‘pre‐ACLF’ group that demonstrates the highest mortality rates and levels of systemic inflammation. The PREDICT study also identified bacterial infection and alcohol‐related hepatitis as main precipitants of ACLF, with the number of precipitants correlating with severity of ACLF and systemic inflammation.^3^

In this issue of the *United European Gastroenterology Journal*, Zanetto et al. introduced the Padua model 2.0 that identifies patients with AD at a high risk of developing ACLF.^4^ This expands on the authors' previous work on the original Padua model, which combined C‐reactive protein (CRP), Chronic Liver Failure Consortium AD score (CLIF‐C AD) and Child Pugh score to predict risk of ACLF in patients with AD, and this model could more accurately predict ACLF development than Model for End‐Stage Liver Disease (MELD), CLIF‐C AD or Child Pugh scores alone.^5^ The Padua model 2.0 differs in that CRP is replaced by presepsin (PSP), a soluble fragment of CD14, as the authors found that high levels of PSP characterised patients with AD.

PSP's superiority in predicting ACLF development likely stems from its specificity to bacterial infection. PSP is released from CD14 during inflammatory responses, where CD14 and its co‐receptor toll‐like receptor 4 (TLR4) on macrophages and monocytes recognise bacterial endotoxin. This specificity probably makes PSP a more accurate predictor of ACLF compared to the hepatically produced acute‐phase protein, CRP.

While PSP results from monocyte‐related inflammation, it is interesting that the latter has been investigated as a potential novel therapy for ACLF. Indeed, in an interplay between lipid metabolism and circulating immunity, dysregulation of the lysophosphatidylcholine and lysophosphatidic acid in ACLF leads to a proinflammatory monocyte phenotype and participates in the immune dysfunction that characterised the syndrome.^6^ This pathway has therefore been proposed to therapeutically reprogramme monocytes, and future research should elucidate its impact on CD14‐related inflammation and ACLF development.

The aim of predicting patients who will develop ACLF is that there would be earlier implementation of interventions that improve survival. In this study, the median time from AD to the onset of ACLF was 66 days leaving enough time to treat and prevent any infections or other precipitants but also, in the promising field of therapeutical approaches to prevent and treat ACLF, to refer patients to clinical trials investigating potential disease‐modifying therapies.^7^ This study clearly highlights the need for the development of preventative therapies for this specific group of patients and the Padua 2.0 model could help to define inclusion criteria for future trials.

These patients could also certainly be referred earlier for a liver transplant assessment but without developing ACLF, they would not receive priority for transplantation under current European transplant listing policies due to MELD not reaching the threshold of the median MELD at transplant. However, placing such patients on waiting lists before its occurrence is probably one of the best ways to secure access to LT during ACLF. A European multi‐centre study has indeed demonstrated that 100% of patients listed before ACLF occurrence were finally transplanted as opposed to 71% of patients listed during the ACLF episode.^8^

In summary, both Padua and Padua 2.0 models are accurate predictive models for identifying patients with AD at high risk of developing ACLF. While both need to be validated in external cohorts, their potential and critical benefits include earlier referral to transplant centres and recruitment in future clinical trials, which should focus on the development of preventative therapies for this group of patients.

## CONFLICT OF INTEREST STATEMENT

The authors declare no conflicts of interest related to this work.

## FUNDING INFORMATION

NIHR; EASL Joan Rodes Fellowship.

## Data Availability

Data sharing not applicable to this article as no datasets were generated or analysed during the current study.
